# A Tuning Fork Gyroscope with a Polygon-Shaped Vibration Beam

**DOI:** 10.3390/mi10120813

**Published:** 2019-11-25

**Authors:** Qiang Xu, Zhanqiang Hou, Yunbin Kuang, Tongqiao Miao, Fenlan Ou, Ming Zhuo, Dingbang Xiao, Xuezhong Wu

**Affiliations:** College of Intelligence Science and Engineering, National University of Defense Technology, Changsha 410073, China; xuqiang09@nudt.edu.cn (Q.X.); kuangyunbin16@nudt.edu.cn (Y.K.); miaotongqiao16@nudt.edu.cn (T.M.); oufenlan15@nudt.edu.cn (F.O.); zhuoming@nudt.edu.cn (M.Z.); dingbangxiao@nudt.edu.cn (D.X.)

**Keywords:** tuning fork gyroscope, spindle azimuth, vibration beam, bias instability, angular random walk

## Abstract

In this paper, a tuning fork gyroscope with a polygon-shaped vibration beam is proposed. The vibration structure of the gyroscope consists of a polygon-shaped vibration beam, two supporting beams, and four vibration masts. The spindle azimuth of the vibration beam is critical for performance improvement. As the spindle azimuth increases, the proposed vibration structure generates more driving amplitude and reduces the initial capacitance gap, so as to improve the signal-to-noise ratio (SNR) of the gyroscope. However, after taking the driving amplitude and the driving voltage into consideration comprehensively, the optimized spindle azimuth of the vibration beam is designed in an appropriate range. Then, both wet etching and dry etching processes are applied to its manufacture. After that, the fabricated gyroscope is packaged in a vacuum ceramic tube after bonding. Combining automatic gain control and weak capacitance detection technology, the closed-loop control circuit of the drive mode is implemented, and high precision output circuit is achieved for the gyroscope. Finally, the proposed Micro Electro Mechanical Systems (MEMS) gyroscope system demonstrates a bias instability of 0.589°/h, an angular random walk (ARW) of 0.038°/√h, and a bandwidth of greater than 100 Hz in a full scale range of ± 200°/s at room temperature.

## 1. Introduction

The Micro Electro Mechanical Systems (MEMS) gyroscope has had great importance in all countries over the past 20 years due to its small size, low cost, low power consumption, and batch fabrication. Micromachined gyroscopes have been widely used in the fields of electronics consumer goods, vehicle safety, weaponry, and other fields [1,2,3]. A significant amount of research has been carried out on the different kinds of micromachined gyroscopes over the past decades, which has directly promoted the development of high performance gyroscopes for various applications. Among the different kinds of MEMS gyroscopes, the typical structures include turning forks (TFG) [4,5,6,7,8], combs [9,10,11], rings or disks [12,13,14,15], or micro wineglass [16,17,18].

The most common type of MEMS gyroscope design is the TFG, a shuttling proof mass design that can be operated in either mode-matched or mode-split conditions. Matched mode tuning fork gyroscopes achieved near navigation-grade performance in [19], where values of 0.003°/√h angular random walk (ARW) and 0.16°/h bias instability were reported. However, its bandwidth was only 1 Hz, failing most navigation application requirements (usually larger than 100 Hz). On the contrary, a gyroscope working in split mode will have a wider bandwidth, but the mechanical sensitivity is not high. So far, there is no split mode gyroscope that can be compared with the performance of a mode-matched gyroscope. In summary, split mode tuning fork micromachined gyroscopes that can meet the requirements of inertial level are rarely reported.

Therefore, enhancing the sensitivity is important for split mode tuning fork gyroscopes. The application field for gyroscopes is determined by their sensitivity; only those gyroscopes with high sensitivity can be applied in the fields requiring small variation in angular velocity. Additionally, the signal-to-noise ratio (SNR) of the gyroscope system will increase after the sensitivity is improved. When the same intensity signal is output by the system, the overall noise of the system is relatively reduced.

It is known that greater driving amplitude and larger initial capacities can lead to higher SNR as well as sensitivity. However, if the driving amplitude is too large, the vertical component displacements will be so great that the vibration mass will collide with the glass substrate. Therefore, the gap between the vibration structure and glass (initial capacitive gap) must be larger to avoid the collision. However, this larger gap could result in low sensitivity due to the smaller initial capacity. Therefore, the vertical component of driving displacement becomes one of key factors limiting the sensitivity of tuning fork gyroscopes. In order to reduce the restriction, the vertical component of the driving displacement must be decreased. However, the vertical component of the driving displacement is related to the spindle azimuth of the vibration beam. Therefore, reasonable design of the cross-section of the vibration beam could improve the spindle azimuth, so as to enhance the sensitivity of the tuning fork gyroscope.

The vibration beam of the micromachined tuning fork gyroscope in our research group is a parallelogram beam, which is fabricated by wet etching [20,21]. Driven by the vertical driving force, the vibration structure generates both horizontal and vertical displacements caused by the parallelogram vibration beam. As there is no structure with a high aspect ratio, this kind of gyroscope structure can be fabricated only by anisotropic wet etching process on a single crystal silicon chip, without advanced dry etching process equipment [22,23]. The processing technology is simple and the cost is low due to the low price of the materials used. However, the spindle azimuth of the parallelogram beam is no larger than 54.74°. Therefore, the sensitivity of the gyroscope is low due to the small spindle azimuth being limited by wet etching process.

A novel silicon bulk gyroscope was proposed by the Sensonor company [24,25,26]. The overall size of the gyroscope structure is very small, benefiting from the unique processing technology, which combines the dry and wet etching. The cross-section of the vibration beam is an irregular polygon, whose spindle azimuth was designed by changing the processing parameters, so the sensitivity of the gyroscope was enhanced by increasing the spindle azimuth. However, as for the dry etching process, it has a strong dependence on advanced processing equipment, and small structure processing requires Silicon-On-Insulator (SOI) chips, which makes the fabrication process more complex and costly than for single crystal silicon chips.

A vibration beam adopting a polygonal cross-section was proposed in order to enhance sensitivity and robustness [27]. Comparison between the proposed polygonal vibration beam and the convex vibration beam on the tolerance capacity analysis was carried out. After comparison, the conclusion was made that the polygonal oblique beam had better tolerance for fabrication imperfections than the convex oblique beam.

Taking all the different kinds of MEMS gyroscopes mentioned above into consideration, in this paper, a tuning fork gyroscope with a polygon-shaped vibration beam is presented. Based on a single crystal silicon chip, the structure is manufactured using processing technology that combines dry and wet etching. Therefore, the following benefits can be obtained:

1. The spindle azimuth can be increased by designing the cross-section of the vibration beam. Therefore, the proposed structure with the optimized vibration beam not only increases the driving amplitude, but also reduces the initial capacitance gap in the sensing direction, so as to enhance the signal-to-noise ratio (SNR) as well as the sensitivity of the gyroscope.
2. The polygonal vibration beam fabricated by the wet and dry etching is much more stable and much more insensitive to the fabrication imperfections, which provides the tuning fork gyroscope with good robustness and repeatability.
3. The use of single crystal silicon chips instead of SOI chips reduces the complexity and cost of the fabrication process.

## 2. Materials and Methods Design for the Proposed Gyroscope

The main idea of the design of the vibration structure is to optimize the cross-section of the vibration beam by improving the spindle azimuth, which should reduce the vertical driving amplitude and enlarge the driving amplitude, so as to enhance the SNR and sensitivity of the gyroscope.

### 2.1. Overall Design of the Proposed Gyroscope

**Figure 1:**
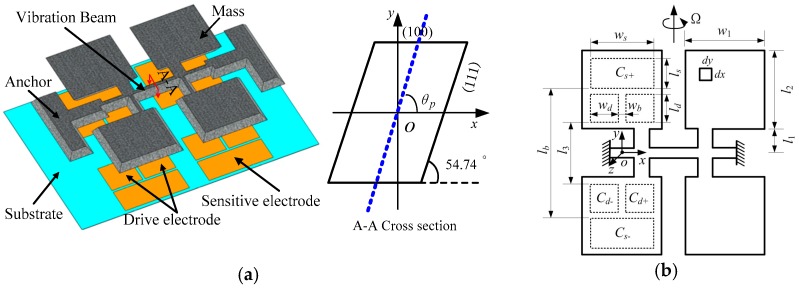
Conventional tuning fork micromachined gyroscope. (**a**) The structure model. (**b**) The structural parameters.

The conventional tuning fork micromachined gyroscope in our work is manufactured by wet etching, and the cross-section of the vibration beam is a parallelogram whose spindle azimuth (*θ_p_*) is no larger than 54.74°, as shown in Figure 1.

Here, *O* is the rotation center of the driving mode, *w_d_* is the width of driving electrode, *w_b_* is the distance between two driving electrodes, *l*_1_ is distance between vibration beam and vibration mass, and *l*_2_ is the length of the vibration mass.

**Figure 2:**
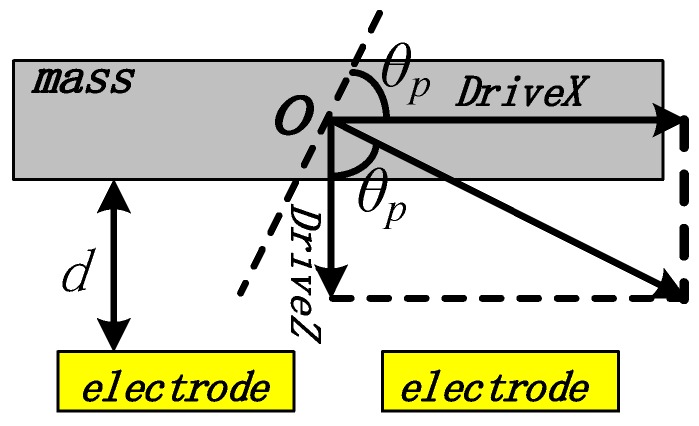
Vertical and horizontal component of driving displacement.

When excited in the driving mode, the vibration mass can generate both horizontal and vertical displacements, as shown in Figure 2. *DriveX* is the horizontal component of driving displacement, *DriveZ* is the vertical component of driving displacement, and d is the initial capacitance gap (*d*).

According to Figure 2, *DriveX* and *DriveZ* can be described as:

(1) $$\left\{ \begin{array}{l} DriveX=\phi_{d}\cdot \sin(\theta_{p})\cdot (l_{1}+l_{2})/2\\ DriveZ=\phi_{d}\cdot \cos(\theta_{p})\cdot (w_{d}+w_{b})/2 \end{array}\right.$$

The maximum vertical component of driving displacement (*MaxdriveZ*) is *d*/3, due to the pull-in effect. Therefore, the maximum horizontal component of the driving displacement (*MaxdriveX*) can be described as:

(2) $$MaxdriveX=(d/3)\cdot \tan(\theta_{p})\cdot (l_{1}+l_{2})/(w_{d}+w_{b})$$

From Equation (2), we can see that if the other parameters are constant, *MaxdriveX* will become larger as the *θ_p_* grows larger.

**Figure 3:**
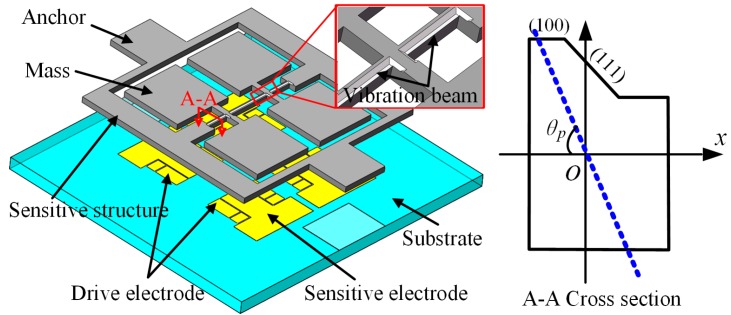
Proposed gyroscope with optimized vibration beam.

In order to enlarge the driving amplitude and enhance the SNR, the cross-section of the vibration beam is designed to increase the spindle azimuth. The proposed structure is fabricated by both wet and dry etching, as shown in Figure 3. The cross-section is an irregular polygon whose spindle azimuth could be designed by changing processing parameters.

### 2.2. Impact Factors of the Spindle Azimuth

The spindle azimuth of the vibration beam is related to the driving amplitude, and it can be increased by optimizing the cross-section shape of the vibration beam.

**Figure 4:**
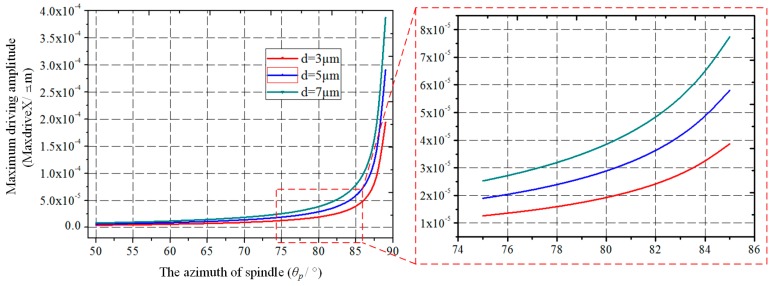
The maximum horizontal component of the driving displacement (*MaxdriveX*) varies with the spindle azimuth (*θ_p_*) and initial capacitance gap (*d*).

According to Equation (2), the *MaxdriveX* is plotted under different gaps (*d*). As can be seen in Figure 4, the *MaxdriveX* grows with the increase of *θ_p_* and gap (*d*). The closer *θ_p_* is to 90°, the faster the *MaxdriveX* increases, especially when *θ_p_* is greater than 80°. Therefore, in order to improve the driving amplitude and enhance the SNR, a larger *θ_p_* should be chosen in the following design.

As the spindle azimuth (*θ_p_*) and initial gap (*d*) grow, the *MaxdriveX* will be enhanced. However, the driving voltage required to drive the vibration mass will be much higher, resulting from the mechanical characteristics of vibration beams with a large spindle azimuth.

According to the research our previous research, the *AC* voltage (*V_ac_*), which is required to drive the vibration mass to *d*/3 in the vertical displacement, can be described as [27]:

(3) $$V_{ac}=\frac{I_{d}w_{d}^{2}d^{3}}{3\varepsilon l_{y}X_{d}A_{d}V_{dc}Q_{d}}\sec^{2}(\theta_{p})$$

where *I_d_* is the moment of inertia of the driving axis, *ω_d_* is the frequency of the driving mode, *d* is the initial capacitor gap between the silicon structure and the glass electrode substrate, *ε* is the electric constant, *l_y_* is the distance from the rotation center to the far end of the mass, *X_d_* is the half distance of the two adjacent driving capacitor centers, *A_d_* is the area of the driving electrodes, *V_dc_* is the *DC* voltage required to drive the structure, and *Q_d_* is the quality factor of the driving mode.

**Figure 5:**
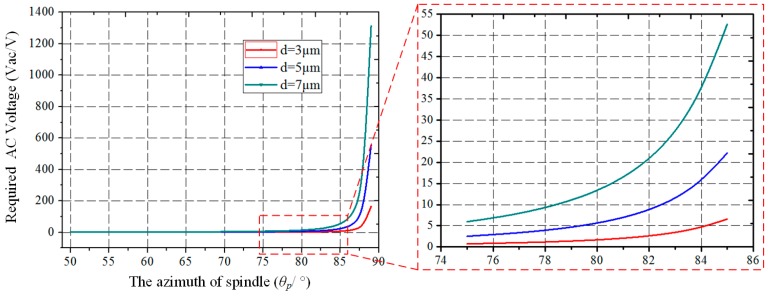
The required AC driving voltage (*V_ac_*) varies with the spindle azimuth.

According to Equation (3), when the other parameters are constant, the relationship between *V_ac_* and *θ_p_* is as shown in Figure 5. As can be seen from the curve, when *θ_p_* is greater than 80°, the required driving *AC* voltage (*V_ac_*) grows quickly, which is difficult when designing a high-voltage circuit.

In the design of the spindle azimuth (*θ_p_*), both the high sensitivity and low driving voltage should be taken into consideration.

### 2.3. Calculation of the Spindle Azimuth

**Figure 6:**
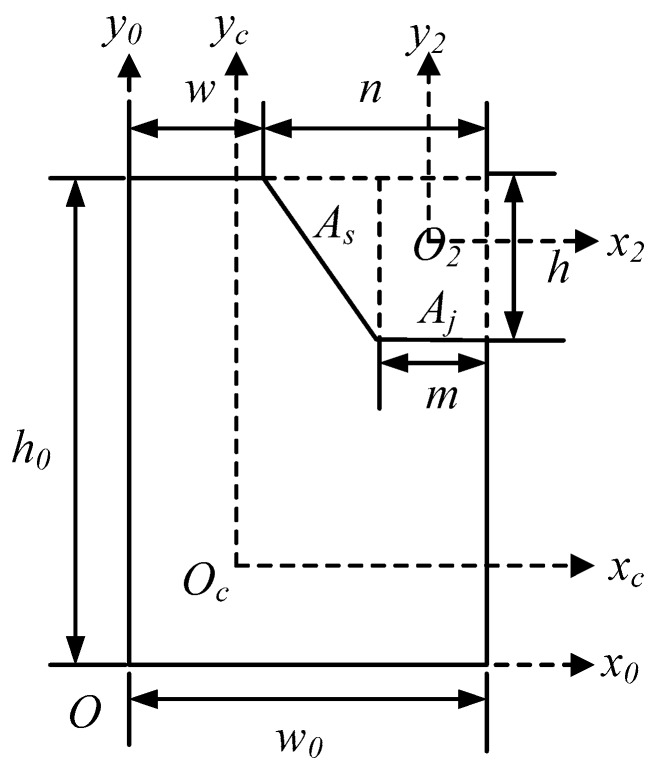
The key dimensions of the cross-section of the proposed vibration beam.

The key dimensions of the cross-section of the proposed vibration beam are shown in Figure 6.

From Figure 6, it can be seen that wet etching is carried out on the basis of a large rectangle, and the right-angled trapezoid part is fabricated by wet etching. Here, *O_c_* is the centroid of the irregular polygon, and *O_2_* is the centroid of the right-angle trapezoid, which consists a right triangle (*As*) and a small rectangle (*Aj*).

Therefore, the spindle azimuth of the proposed vibration beam can be accurately controlled by changing the width (*w*) and depth (*h*) of wet etching.

In order to design the spindle azimuth of the irregular polygon (*θ_p_*), the moments of inertia (*I_x_c_* and *I_y_c_*) and the product of inertia (*I_x_c_y_c_*) of the irregular polygon should be calculated beforehand, after which the spindle azimuth can be calculated and described as [28]:

(4) $$angle=angle=90-\arctan[2\cdot I\_x_{c}y_{c}/(I\_y_{c}-I\_x_{c})]/(2\cdot \pi \cdot 180)=f(w,h)$$

**Figure 7:**
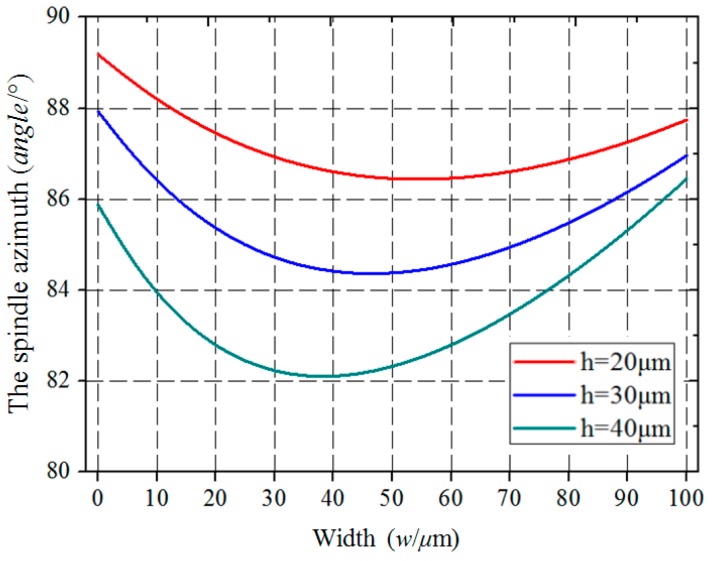
The spindle azimuth (*θ_p_*) varies with the width (*w*) and depth (*h*) of wet etching.

According to Equation (4), we can see that the spindle azimuth of the irregular polygon (*θ_p_*) is affected by the width (*w*) and depth (*h*) of wet etching, as shown in Figure 7.

### 2.4. Design of the Main Dimensions of the Cross-Section 

According to Figure 7, when the other conditions are constant, the spindle azimuth (*θ_p_*) decrease as the depth (*h*) of wet etching deepens, and the spindle azimuth (*θ_p_*) decreases first and then increases as the width (*w*) becomes wider. Therefore, when designing the spindle azimuth (*θ_p_*), the zone around the minimum point on the curve is a better choice due to the insensitivity to dimensional errors of width (*w*).

When the width (*w*) becomes larger, the curves with different etching depths *(h*) become closer. That is to say, the spindle azimuth (*θ_p_*) is insensitive to dimensional errors of wet etching depth (*h*) when the width of wet etching (*w*) becomes larger.

In order to obtain a better spindle azimuth (*θ_p_*) that is less affected by fabrication errors, both the width error and depth error should be considered comprehensively. The different processing parameters for the spindle azimuth are calculated with the same processing errors.

**Figure 8:**
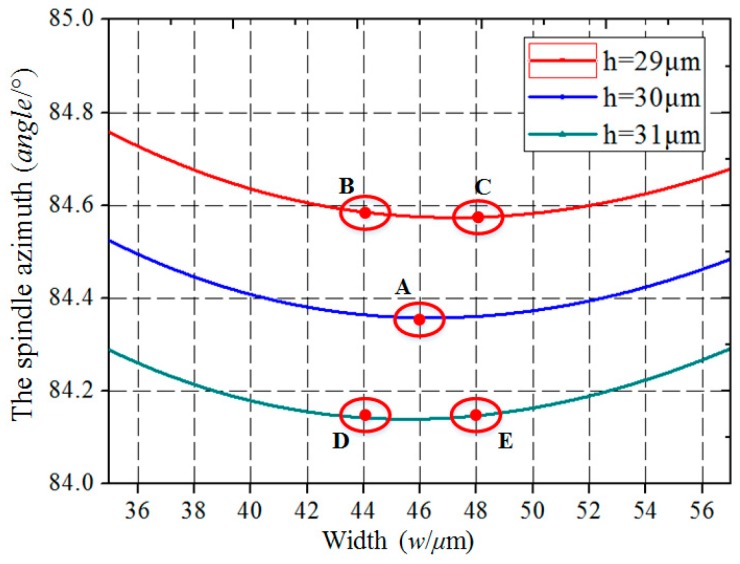
The spindle azimuth (*θ_p_*) varies with the processing errors.

After comparison between the different approaches, the optimized combination of structure parameters is obtained, providing insensitivity to dimensional errors. As can be seen in Figure 8, point A is the ideal design of the spindle azimuth on the second line, and the other four points (B, C, D, and E) are the results with 1 μm error in depth and 2 μm error in width.

**Figure 9:**
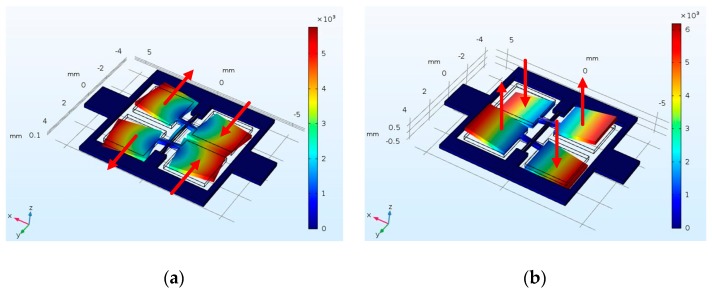
The modal simulation results: (**a**) drive mode (3253.8 Hz); (**b**) sense mode (3414.7 Hz).

The optimized structural parameters of the proposed vibration structure are determined by theoretical analysis, finite element simulation, and experimental verification. Using the finite element analysis method, the modal simulation analysis of the tuning fork gyroscope is carried out in the simulation software COMSOL Multiphysics, and the simulation motion form of the working mode of the gyroscope can be obtained. The simulation results for drive and sense modes are shown in Figure 9.

As can be seen from Figure 9, the drive mode shown in Figure 9a is the bending motion of the vibration beam. The sense mode shown in Figure 9b is the torsional motion of the vibration beam.

#### 2.4.1. Driving Mode

There are a pair of differential drive electrodes and a detection electrode under each vibration mass block, as shown in Figure 3. When driving voltages are applied to the differential drive electrode plates, the driving electrostatic force will be generated. When excited by the driving electrostatic force, bending deformation occurs in the vibration beam, so the vibration mass block oscillates in the horizontal direction. However, because the azimuth angle of the vibration beam is less than 90 degrees, the vertical driving motion component is also generated. Therefore, in the driving mode of the gyroscope, the vibration mass block has a three-dimensional vibration motion, which consists of the same reciprocating motion frequency in the horizontal direction and the driving component motion in the vertical direction, as shown in Figure 9a.

#### 2.4.2. Detection Mode

When the gyroscope is driven in driving mode and there is angular velocity input on the sensitive axis, a Coriolis force will be generated in the direction of detection, according to the principle of Coriolis force. This Coriolis force will cause torsional deformation of the vibration beam, which will cause the vibration mass block to move up and down in the vertical direction, as shown in Figure 9b.

#### 2.4.3. Working Condition

**Figure 10:**
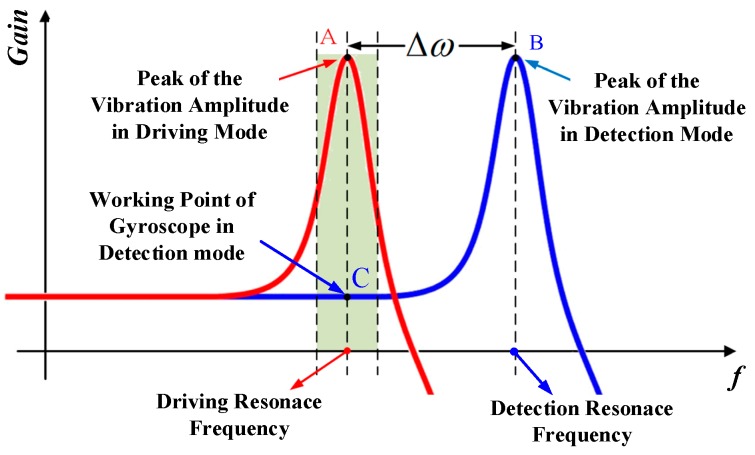
The theoretical amplitude–frequency response curves of the tuning fork gyroscope.

The proposed tuning fork gyroscope uses the frequency mismatch model, and its working frequency is the driving mode frequency. The amplitude–frequency response curves of the working modes of the gyroscope can be obtained by frequency response analysis (FRA). The theoretical amplitude–frequency response curves of the tuning fork gyroscope are shown in Figure 10.

As can be seen in Figure 10, points A and B are the peaks of the vibration amplitude in driving and detection curves, respectively. The frequencies of point A and point B are the driving and detection resonance frequencies of the gyroscope. The frequency difference between A and B is defined as the frequency difference of the tuning fork gyroscope. Crossing point C instead of detection resonance frequency point B is the working point of the gyroscope in detection mode. This is why gyroscopes operating in mismatched mode have lower sensitivity but higher stability. 

Therefore, in the optimization design of the tuning fork gyroscope, it is necessary to select the operating frequency difference of the gyroscope reasonably. On the premise of ensuring the stability of the working state of the gyroscope, it is of great significance to reduce the frequency difference as much as possible in order to obtain the maximum sensitivity, so as to improve the performance of the gyroscope.

## 3. Fabrication Processing Technology

The fabrication process consists of silicon structure processing, glass electrode substrate processing, bonding processing, and packaging processing. 

### 3.1. Fabrication Processing of Silicon Structure and Glass Electrode Substrate

**Figure 11:**
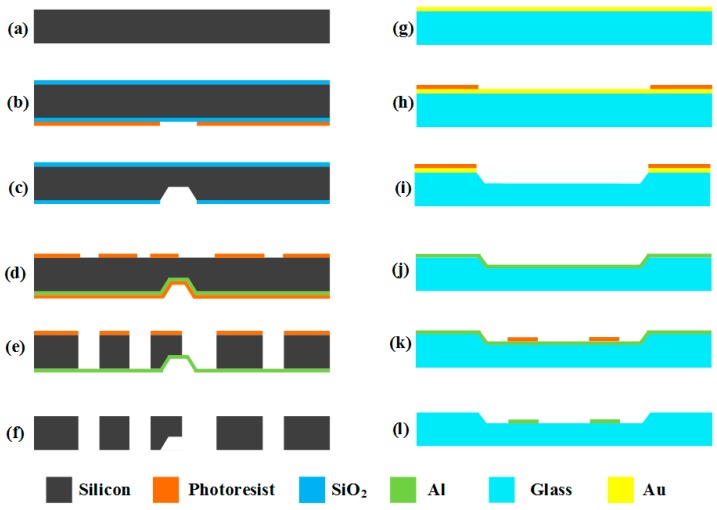
Fabrication process of the silicon structure and glass electrode substrate: (**a**) The processing of the structure starts with a double polished silicon wafer. (**b**) After high-temperature thermal oxidation, a layer of SiO_2_ is grown on the silicon wafer, and then photolithography and development processes are done on the back side of the silicon wafer. Part of the photoresistant layer is removed to prepare for the wet etching in the next step. (**c**) After removing part of SiO_2_, the remaining SiO_2_ is used as a new mask, and then the wet etching process is conducted on the silicon wafer. (**d**) Firstly, the SiO_2_ on both sides is removed, and a layer of aluminum is sputtered on the back side of the silicon wafer as the cut-off layer. Then, a layer of photoresistant layer is used to protect the aluminum from the corrosion of the developer. At last, the second photolithography and development processes are done on the front side of the silicon wafer. Part of photoresistant layer is removed to prepare for the dry etching in the next step. (**e**) After removing the photoresistant protective layer on the back, dry etching is done on the front side continuously until the whole silicon wafer is etched to the cut-off layer. (**f**) Removing all of the photoresistant layer and the aluminum, the structure processing is completed. (**g**) The processing of the electrode substrate starts with a glass wafer, and a layer of gold is evaporated on the front of the glass wafer. (**h**) The photolithography and development processes are done on the gold layer, and part of photoresistant layer is removed. (**i**) After removing part of the Au, the remaining Au serves as the mask, and wet etching is done on the glass wafer to make the gaps and provide the motion space for the silicon structure (c). (**j**,**k**) After removing all of the photoresistant layer and the Au, a layer of aluminum is sputtered on the gap. Then, the photolithography and development processes are done on the aluminum layer. Part of photoresistant layer is then removed, and the rest of the photoresistant layer serves as the mask to prepare for the next step. (**l**) After removing the aluminum without mask layer protection, the mask layer is removed and the electrode substrate is finished.

The fabrication processing of the silicon structure, which combines wet and dry etching, can be seen in Figure 11a–f. The fabrication processing of the glass electrode substrate can be seen in Figure 11g–l.

### 3.2. Bonding and Packaging Process

**Figure 12:**
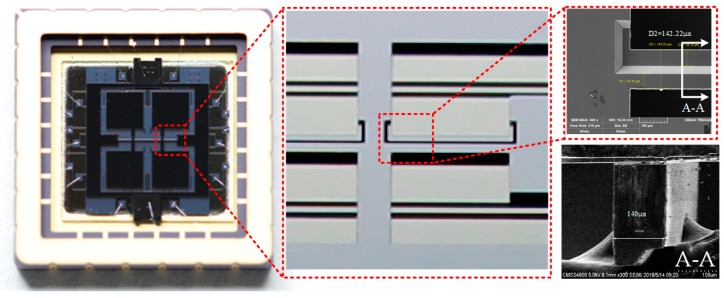
Proposed gyroscopes were packaged in ceramic tubes.

The structure and electrode substrate are bonded together with a narrow capacitive gap, which can increase the electromechanical coupling. After bonding, the gyroscopes are packaged in a ceramic chip carrier in a vacuum environment in order to minimize the Brownian noise floor to achieve better performance. The entire device can be observed under an optical microscope, and some parts of the structure can be observed by scanning electron microscope (SEM), as shown in Figure 12.

## 4. Performance Testing

The performance testing of gyroscopes in ceramic tubes mainly includes modal testing and zero bias testing.

### 4.1. Circuit Signal Processing Technology

There is a detection electrode and a pair of driving electrodes below each vibration mass. When electrodes with the same phase are connected together, the microelectromechanical gyroscope is equivalent to a five-terminal device [29].

**Figure 13:**
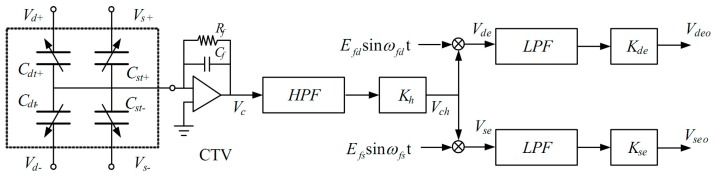
The equivalent model of the gyro circuit.

As can be seen in Figure 13, the device consists of four capacitors and one common terminal, which is the silicon structure of the gyroscope. *C_dt+_* and *C_dt−_* are the differential capacitances for the driving axis, while *C_st+_* and *C_st−_* are the differential capacitances for the sensing axis. Here, *V_d+_* and *V_d−_* are applied to the driving electrodes, while *V_s+_* and *V_s−_* are applied to the sensing electrodes. (Where, CTV stands for capacitance/voltage converting, HPF stands for high pass filter and LPF stands for low pass filter.)

The voltages applied to the differential capacitance are shown in the following equation:

(5) $$\left\{ \begin{array}{l} V_{d+}=V_{dc}+V_{ac}\sin\omega_{d}t+E_{fd}\sin\omega_{fd}t\\ V_{d-}=V_{dc}-V_{ac}\sin\omega_{d}t-E_{fd}\sin\omega_{fd}t\\ V_{s+}=E_{fs}\sin\omega_{fs}t\\ V_{s-}=-E_{fs}\sin\omega_{fs}t \end{array}\right.$$

where *V_dc_* is the DC bias, *V_ac_*sin*ωdt* is the AC drive voltage, *E_fd_*sin*ω_fd_t* and *E_fs_*sinω*_fs_t* are the high frequency carriers in the driving and sensing axes. All the high frequency carriers come from the pre-set output of the Microcontroller Unit (MCU). So, the output voltage of the charge amplifier (*V_c_*) can be described as:

(6) $$V_{c}=-(V_{d+}C_{dt+}+V_{d-}C_{dt-}+V_{s+}C_{st+}+V_{s-}C_{st-})/C_{f}$$

After passing through the high pass filter (*HPF*) and amplification (*K_h_*), the output voltage *V_ch_* can be described as:

(7) $$V_{ch}=-(K_{h}E_{fd}\sin\omega_{fd}t\Delta C_{d})/C_{f}-(K_{h}E_{fs}\sin\omega_{fs}t\Delta C_{s})/C_{f}$$

After passing through the multiplier, the signal *V_ch_* is demodulated by the high-frequency driving and sensing carriers, respectively. So, the end output voltages *V_deo_* and *V_seo_* can be described as:

(8) $$\left\{ \begin{array}{l} V_{deo}=-(K_{h}K_{de}E_{fd}^{2}\Delta C_{d})/2C_{f}\\ V_{seo}=-(K_{h}K_{de}E_{fs}^{2}\Delta C_{s})/2C_{f} \end{array}\right.$$

From Equation (8), we can see that the output voltages *V_deo_* and *V_seo_* are proportional to the variation of the driving and sensing capacitance, respectively. Thus, the variation of the driving and sensing capacitance can be effectively extracted and separated.

**Figure 14:**
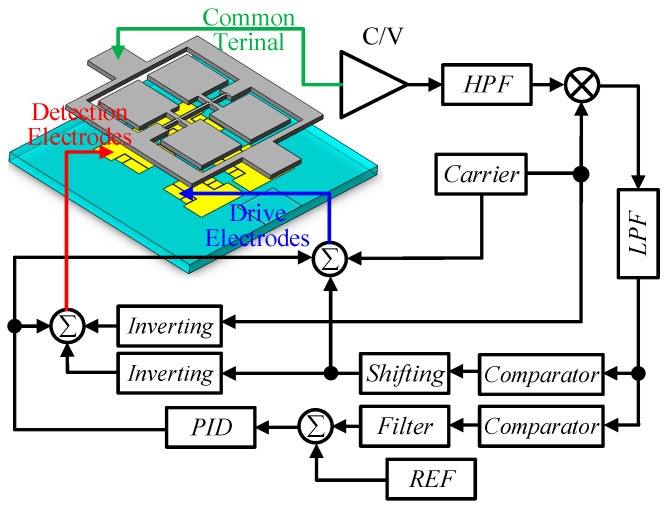
The schematic diagram of the closed driving loop control.

In the closed driving loop, automatic gain control (AGC) technology is used to ensure the stability of the driving voltage. The high frequency carrier signal of the driving voltage is applied to modulate the signal of the high frequency carrier signal. Then, the output signal on the common terminal is demodulated with this carrier signal, and the information for the driving loop can be obtained. The schematic diagram of the closed driving loop control is shown in Figure 14. (Where, C/V stands for capacitance/voltage converting, PID stands for Proportion Integral Differential and REF stands for reference.)

In order to separate the detection loop information from the common terminal and reduce the influence of low-frequency noise, the high-frequency detection carrier is applied to the detection electrodes. The detection capacitance variation signal is modulated to the high frequency of the carrier signal, and the useful signal and the low-frequency noise are successfully separated. 

**Figure 15:**
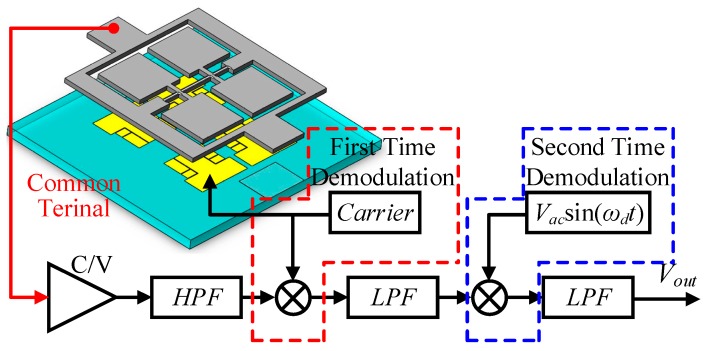
Schematic diagram of weak capacitance detection.

The first demodulation with the high-frequency carrier separates the useful signal from other signals at the common terminal. The frequency of the obtained signal is the frequency of the applied AC driving voltage. The output signal after first demodulation is demodulated by the AC driving voltage for the second time, and the DC voltage signal corresponding to the input angular velocity is obtained, as shown in Figure 15. 

### 4.2. Modal Testing

**Figure 16:**
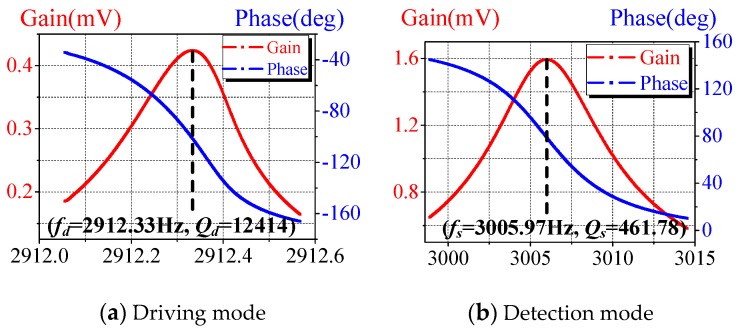
Modal testing of the proposed gyroscope.

Frequency response analysis (FRA) is used to perform modal testing of the proposed gyroscope, and the results are shown in Figure 16. The modal testing is an important part of the performance testing. On the one hand, the natural vibration frequencies of driving and detection modes can be obtained, and the working state of the gyroscope can be accurately detected. On the other hand, the quality factor (Q) of the driving and detection modes can be calculated according to the modal test curves, and the vacuum situation of the gyroscope can be obtained, which provides an important reference for subsequent performance testing.

According to the characteristics of the micro-gyroscope proposed in this paper, a method combing electrostatic force excitation and capacitance detection is used for modal testing. A frequency response analyzer is used to apply sweep excitation on driving and detection electrodes, and the response of the gyro to the signal is observed. The modal frequencies and Q values are obtained from the frequency response analysis curves. The frequency response analyzer is a FRA5087 from Negative Feedback (NF) company. Its frequency resolution is 0.1 MHz, which meets the test requirements for the gyroscope [30].

The quality factor of the gyroscope is calculated in the –3dB bandwidth. The frequency response in the driving direction is shown in Figure 16a, the resonance frequency is 2912.33 Hz, and the quality factor is 12,414. The frequency response in the detection direction is shown in Figure 16b, the resonance frequency is 3005.97 Hz, and the quality factor is 461.78.

### 4.3. Zero Bias Testing

**Figure 17:**
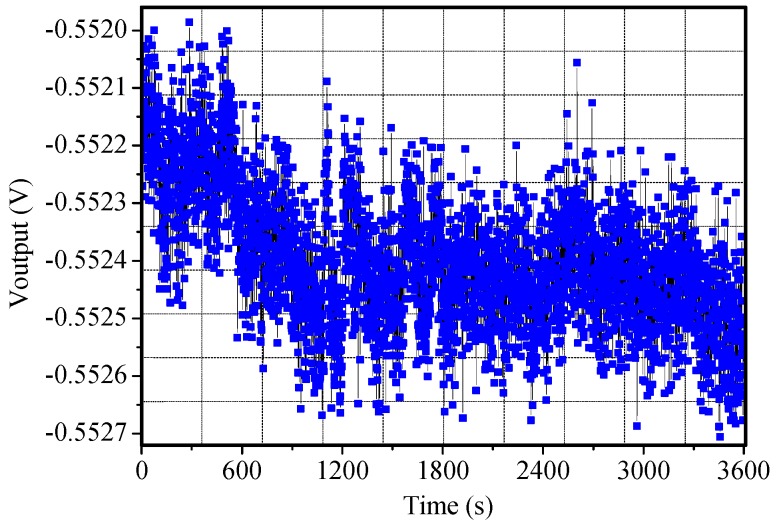
Zero bias output of the sample.

The zero bias testing is measured at room temperature, and the bias stability of sample reaches about 1.3°/h, as shown in Figure 17. 

**Figure 18:**
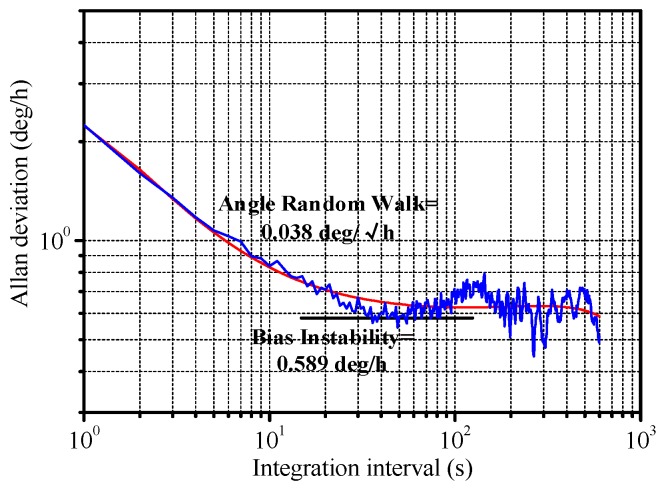
Allan deviation of the sample.

Allan variance is used to analyze the bias stability. The standard definition of bias instability used by inertial sensor manufacturers is the minimum point on the Allan variance curve. The system demonstrates a bias instability of 0.589°/h and an angular random walk (ARW) of 0.038°/√h, as shown in Figure 18.

As can be seen from the performance results, compared to other reported high-performance MEMS gyroscope, the performance of the proposed tuning fork gyroscope is not good enough. The noise level of the gyroscope is critical to the performance, and the low noise level of the gyroscope is one of the reasons for its poor performance. So, the analysis of gyroscope noise is carried on, which is helpful for performance improvement.

## 5. Noise Analysis

The resolution and minimum bias stability of the MEMS gyroscope are determined by the noise level of the angular velocity output signal. The noise of the gyroscope system consists of the mechanical thermal noise from the silicon structure and the circuit noise from the detection circuit.

### 5.1. Mechanical Thermal Noise

Mechanical thermal noise is the main noise resource of the MEMS gyroscope. It is caused by irregular thermal motions of gas molecules around inertial mass and molecules inside the structure, and is directly related to the damping of the system. The greater the damping of system, the greater the mechanical thermal noise. The equivalent force of the mechanical thermal noise in the MEMS gyroscope can be described as [31]:

(9) $$F_{mtn}=\sqrt{4k_{B}TcB}$$

where *k_B_* is the Boltzmann constant, *T* is the absolute temperature in degrees Kelvin, *c* is the damping coefficient of the system, and *B* is the bandwidth of the gyroscope. 

The equivalent angular velocity of the mechanical thermal noise in the gyroscope in this paper can be described as:

(10) $$\Omega_{TMN}=\frac{1}{x_{0}}\sqrt{\frac{k_{b}T\omega_{s}B}{m\omega_{d}^{2}Q_{s}}}$$

where *x*_0_ is the dynamic displacement along the driving direction, *ω_d_* and *ω_s_* are the resonant frequencies of driving and detection modes, *m* is the equivalent vibration mass, and *Q_s_* is the quality factor of the detection mode. According to Equation (10), reducing the mechanical thermal noise of the gyroscope so as to improve the performance can be achieved by improving the driving displacement and equivalent vibration mass and increasing the *Q_s_*.

### 5.2. Noise in the Detection Circuit

**Figure 19:**
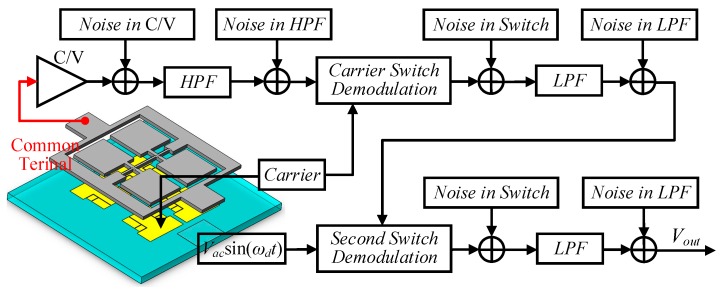
The main noise sources of the gyroscope in detection circuit.

The minimum signal level that a circuit can handle correctly is determined by the noise of the detection circuit. The greater the noise of the circuit, the poorer the ability of the circuit is to detect or process small signals, and the poorer the performance of the gyroscope system. In order to analyze the circuit noise of the gyroscope, the noise model of the gyroscope system needs to be established. The circuit noise of the gyroscope is mainly related to the detection circuit, so the noise analysis in the detection circuit is focused on this. The main noise sources of the gyroscope in the detection circuit can be seen in Figure 19.

As can be seen in Figure 19, the switch demodulation is conducted at a high frequency, so the noise generated by the switch is restrained after passing through the low pass filter. The charge amplifier is located at the front end of the detection circuit, so the noise generated by the charge amplifier is the main circuit noise. 

**Figure 20:**
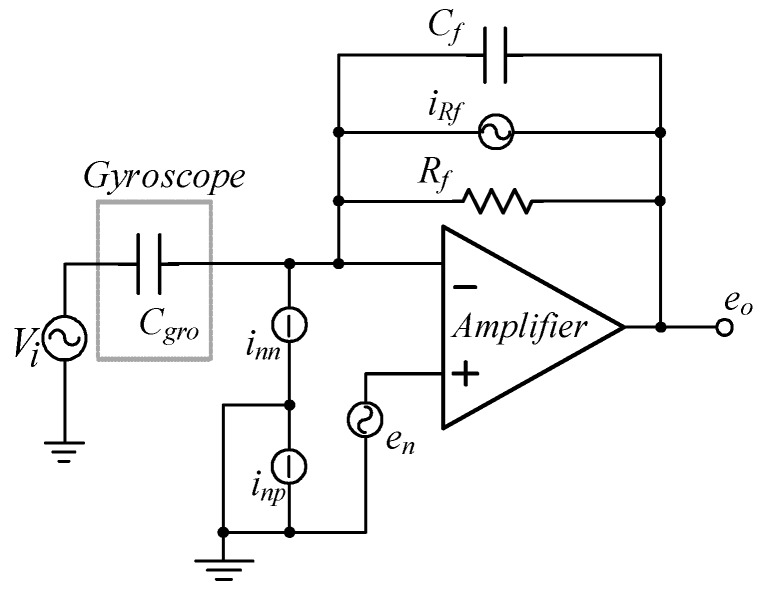
The noise model of the front-end charge amplifier in the detection circuit.

The noise model of the front-end charge amplifier is established in Figure 20. 

The noise of the front-end charge amplifier mainly includes three parts: equivalent input voltage, circuit noise, and resistance thermal noise. Capacitors do not produce noise, but instead limit the frequency bandwidth of the output voltage. Therefore, the total output noise of the charge amplifier is contributed by the operational amplifier and resistor.

Because of the irrelevance of the noise, the total power density of the output noise can be calculated as:

(11) $$e_{oc}^{2}=e_{n}^{2}{\left(1+\frac{C_{gro}}{C_{f}}\right)}^{2}+\left(i_{nn}{}^{2}+\frac{4kT}{R_{f}}\right){\left|\frac{1}{{\left(j\omega C_{f}\right)}^{2}}\right|}^{2}$$

where *C_gro_* is the sum of the driving voltage and detection capacitance of the gyroscope, *C_f_* is the feedback capacitance of the charge amplifier, and *R_f_* is the feedback resistor of the charge amplifier.

If *e_oc_* is equivalent to capacitive noise density *c_nc_*, then *e_oc_* can be described as:

(12) $$c_{nc}^{2}=e_{n}^{2}\frac{{\left(C_{f}+C_{gro}\right)}^{2}}{E_{fs}^{2}}+\left(i_{nn}^{2}+\frac{4kT}{R_{f}}\right)\left|\frac{1}{{\left(j\omega_{fs}\right)}^{2}E_{fs}^{2}}\right|$$

According to Equation (12), the main measures to reduce circuit noise are as follows:

(a) Select a better operational amplifier with lower noise *e_n_*.
(b) Increase the frequency of carrier *ω_f2_*.
(c) Decrease the feedback capacitor of the charge amplifier *C_f_*.
(d) Improve the amplitude of carrier *E_f2_*.

## 6. Conclusions

The design, simulation, fabrication, and experimentation of a novel split-mode, micromachined tuning fork gyroscope has been presented in this paper. Increasing the spindle azimuth of the vibration beam of the gyroscope can not only improve the driving displacement, but can also reduce the vibration gap and enhance the SNR of the sensor. The impact factors of the spindle azimuth of the vibration beam were designed and analyzed. The fabrication processes were demonstrated, including the manufacture of the vibration structure, glass substrate, anodic bonding, and vacuum packaging process. Finally, performance testing of the gyroscope in a ceramic tube was conducted. The zero-rate bias instability and ARW were obtained as 0.589°/h and 0.038°/√h from measurements at room temperature, respectively. 

After analyzing the noise of the tuning fork gyroscope, there is still a lot of work to do. On the one hand, it is necessary to improve the processing quality to reduce the mode coupling and zero bias. On the other hand, a better operational amplifier with lower noise should be applied to improve the overall signal-to-noise ratio of the gyroscope and further improve the performance of the gyroscope.
